# Short communication: performance of beef cows fed Kernza intermediate wheatgrass straw mixed with alfalfa haylage

**DOI:** 10.1093/tas/txaf131

**Published:** 2025-12-02

**Authors:** Dante M Pizarro, Arin Crooks, Michel A Wattiaux, Valentin D Picasso

**Affiliations:** Department of Plant and Agroecosystem Sciences, University of Wisconsin-Madison, Madison, WI 53706, United States; Lancaster Agricultural Research Station, University of Wisconsin-Madison, Lancaster, WI 53813, United States; Department of Animal and Dairy Sciences, University of Wisconsin-Madison, Madison, WI 53706, United States; Department of Plant and Agroecosystem Sciences, University of Wisconsin-Madison, Madison, WI 53706, United States; Department of Crop Production Ecology, Swedish University of Agricultural Sciences, Ulls väg 16, Uppsala, 75651, Sweden

**Keywords:** cattle, perennial, crop by-product

## Abstract

Kernza intermediate wheatgrass (*Thinopyrum intermedium* [Host] Barkworth & D.R. Dewey) is a novel dual-use perennial grain and forage crop with environmental and economic benefits for farmers. Perennial crop byproducts, such as Kernza straw, have been suggested as an alternative forage source in livestock systems. However, there is limited information on cattle performance offered Kernza straw. Therefore, our study assessed the performance of mature beef cows fed Kernza straw mixed with a haylage composed of alfalfa (*Medicago sativa* L.) and cool-season grasses. Grasses included orchardgrass (*Dactylis glomerata* L.), meadow fescue (*Festuca pratensis* L.) and Kentucky bluegrass (*Poa pratensis* L.). Two feeding trials conducted in two different years were performed with 36 pregnant Angus cows (*Bos taurus*) in six pens of six animals each in a completely randomized design with three replications. Dietary treatments included: (i) a 100% grass-alfalfa haylage (control), and (ii) a 50% Kernza straw − 50% grass-alfalfa haylage. Average daily gain was lower in the Kernza straw cows than in the control group (0.41 vs 0.92 kg day^−1^) in Trial 1 (*P *= 0.02) with no differences in Trial 2 (*P *= 0.13). Daily dry matter intake did not change in Trial 1 (*P *= 0.08), while for the cows offered Kernza straw it was reduced from 12.9 to 11.3 kg cow^−1 ^day^−1^ in Trial 2 (*P* < 0.01). There were no changes in body condition among cows fed different diets in both trials (*P *> 0.05). Therefore, 50% Kernza straw can be successfully used in beef cow diets at least for 60 days without negative impacts on animal performance and potential economic and environmental benefits.

## Teaser

- Pregnant beef cows did not lose weight or reduce their body condition with a diet containing 50% Kernza straw.

## Introduction

Management practices like growing annual crops (e.g., corn and sorghum as feed grain) and fertilization in beef cattle operations can increase environmental impacts such as greenhouse gas emissions or polluting water resources (Stewart et al. 2005; Tilman et al. 2002). Additionally, these practices drive up operational costs, with feed accounting for up to 75% of a beef cattle farm’s annual expenses (USDA 2022). In contrast to annual crops, perennial grain crops represent a paradigm shift in modern agriculture as they can significantly reduce the use of expensive inputs and the environmental and operational costs of tillage, planting, and fertilization (Chapman et al. 2022; Picasso et al. 2022). Intermediate wheatgrass (*Thinopyrum intermedium* [Host] Barkworth & D.R. Dewey) is a cool-season perennial grass grown, harvested, and sold for both grain and forage across the USA (Lanker et al. 2020). Kernza is the trademark name for its grain. Currently, Kernza is grown in 2404 acres by 95 farmers in the USA (The Land Institute 2024). Production of Kernza summer forage or straw ranged from 3.2 to 11.8 t ha^−1^ across different US states (Franco et al. 2021). Therefore, straw is a relevant co-product of Kernza grain production, with production costs partially covered by the grain enterprise. Expanding its use in beef diets represents an opportunity for beef producers to decrease feeding costs and increase the profitability of their operations.

The primary limitation of using Kernza straw is the low forage nutritive value (Favre et al. 2019). Recent studies have reported the concentration of neutral detergent fiber (NDF), acid detergent fiber (ADF), and crude protein (CP) in Kernza straw to range from 67.2 to 73.3, 38.2 to 48.9, and 2.7% to 5.6% dry matter (DM), respectively (Culman et al. 2023; Hunter et al. 2020; Pinto et al. 2022). Forages with more than 68% NDF and less than 8% CP often do not allow *Bos taurus* cows to maintain condition, as the high fiber concentration restricts their intake and microbial activity in the rumen is limited by the lack of nitrogen (Moore and Undersander 2001; NASEM 2016). Therefore, an effective way to balance and utilize low-quality forages, like Kernza straw, involves mixing with a greater-quality forage, a strategy commonly implemented with prairie hay or low-quality grass hay (Titgemeyer et al. 2004). However, Kernza straw is a novel product, and no data on the performance of beef cows are yet available. Thus, our objective was to assess the performance of mature beef cows fed Kernza straw mixed with grass-alfalfa haylage. Our hypothesis was that beef cows fed a mix of Kernza straw and grass-alfalfa haylage would maintain their performance compared with those fed only grass-alfalfa haylage.

## Materials and methods

All procedures for this study were approved by the Institutional Animal Care and Use Committee of the College of Agricultural and Life Sciences at the University of Wisconsin-Madison (A006291).

### Experimental design

This feeding trial used a completely randomized experimental design, with two treatments (diets) allocated to each of six pens (three replications) of six cows each. The experiment was replicated over two years, as Trial 1 and Trial 2, with different groups of cows. Trial 1 lasted from 19 December 2017 to 22 February 2018 (65 days) and Trial 2 from 3 March to 29 April 2020 (58 days). Details of treatments, animals, and analyses are described below.

### Diets and nutrient composition

A Kernza field was planted with 12 kg ha^−1^ (pure live seed) of Cycle-4 Kernza seeds from The Land Institute (Salina, KS, USA). Planting was carried out on September 15, 2016 using a Great Plains 1006NT no-till grain drill in the University of Wisconsin-Madison Lancaster Agricultural Research Station, Lancaster, WI, USA with a row spacing of 12.7 cm. In the Spring of 2017, fertilization consisted of a split application of 33.6 kg N ha^−1^ at green up (April 10, 2017) and the same amount after stem elongation (May 12, 2017). In the Spring of 2018, 56 kg N ha^−1^ were applied (March 27). Nitrogen was applied in the form of urea. The straw was harvested on August 1, 2017, and July 26, 2019, as a co-product of the Kernza grain crop. Grain yields were 926, 321 and 122 kg ha^−1^, while straw yields were 6680, 6760, and 4520 kg ha^−1^ in 2017, 2018, and 2019, respectively. The whole plants were swathed at the late dough stage to a stubble height of 10.2 cm and laid in a wind row until the grain was threshed with a combine harvester (Axial-Flow 1688, Case International Harvester, Racine, WI). The straw left on the field after threshing was baled at 87.6% and 85.5% DM concentration in Trials 1 and 2, respectively and stored under roof until it was chopped and stored in a loose pile under roof. The haylage, from a nearby field at Lancaster, was composed of an equal proportion of alfalfa (*Medicago sativa* L.) and cool-season grasses, primarily orchardgrass (*Dactylis glomerata* L.), meadow fescue (*Festuca pratensis* L.) and Kentucky bluegrass (*Poa pratensis* L.), and was chopped and ensiled with a DM concentration of 35.5% and 39.0% in Trials 1 and 2, respectively. Alfalfa and grasses were harvested every 30–35 days, aiming for a development stage of 25% bloom.

The haylage diet consisted of 100% haylage, and the Kernza diet consisted of a mixture of 50% Kernza straw and 50% haylage, mixed in equal proportions by DM weight in a Knight Reel Augie 2250 mixing wagon (Brodhead, WI, USA). Minerals were included in the total mixed ration (TMR).

In Trial 1, forage samples were taken from 10 different locations in the haylage bag and 10 randomly selected Kernza straw bales. Samples from each material were dried and combined into one composite sample for the Kernza straw and two composite samples for the haylage diet. Samples were sent to Dairyland Laboratories (Arcadia, WI, USA). Dried Kernza straw sample was analyzed for DM (NFTA 2001), NDF using α-amylase and sodium sulfite (method 2002.04; AOAC International 2006), ADF (method 973.18, AOAC International, 2000), CP using the Kjeldahl method (method 990.03; AOAC International 2006), and minerals (method 985.01; AOAC International 2000) concentration. Dried haylage samples were analyzed for DM, NDF, ADF, and CP composition by using near-infrared spectroscopy (NIRS). Calibration equations were based on wet chemistry procedures described previously. Nutrient composition of the 50-50 Kernza-haylage diet was determined by the proportion of Kernza straw and grass-alfalfa haylage on the diet. In Trial 2, grass-alfalfa haylage diet and Kernza straw samples were collected weekly, and 50-50 Kernza-haylage diet samples were collected every other week. Samples were frozen, dried, and sent to UW Soil and Forage Analysis Lab (Marshfield, WI, USA). Samples were analyzed for DM, NDF, ADF, and CP through NIRS. Calibration equations were based on wet chemistry procedures described previously. Concentration of total digestible nutrients (TDN) was calculated as 88.9 – (0.779 × % ADF) according to Linn and Martin (1989). Feedstuffs and diet composition are presented in Table 1. Additionally, predictions of dry matter intake (DMI), as well as energy and protein available and required, were calculated using NASEM (2016).

**Table 1.** Nutrient composition parameters (mean ± standard deviation) of feed ingredients (grass-alfalfa haylage or kernza straw) and diet treatments (100% grass-alfalfa haylage and 50% grass-alfalfa haylage + 50% kernza straw) for feeding trials at Lancaster, WI, USA over two years (*n* = number of samples used from each ingredient or diet).

	Trial 1			Trial 2		
Item^a^	Grass-alfalfa haylage diet (*n* = 2)	Kernza straw (*n* = 1)	50-50 Kernza-haylage diet (*n* = 1)	Grass-alfalfa haylage diet (*n* = 7)	Kernza straw (*n* = 7)	50-50 Kernza-haylage diet (*n* = 4)
DM (%, as-fed)	35.5 ± 0	87.6	50.5	39.0 ± 1	85.5 ± 4	53.4 ± 2
NDF (% DM)	60.5 ± 4	73.3	66.9	48.4 ± 7	72.4 ± 2	60.4 ± 3
ADF (% DM)	43.7 ± 1	48.9	46.3	40.1 ± 3	48.5 ± 1	43.8 ± 1
CP (% DM)	10.1 ± 2	5.6	7.9	16.2 ± 2	4.1 ± 1	12.6 ± 1
Calcium (% DM)	0.58 ± 0.16	0.19	0.39	0.86 ± 0.26	0.32 ± 0.02	0.61 ± 0.13
Phosphorus (% DM)	0.23 ± 0.01	0.15	0.19	0.33 ± 0.02	0.15 ± 0.02	0.29 ± 0.01
Magnesium (% DM)	0.25 ± 0.04	0.09	0.17	0.28 ± 0.02	0.15 ± 0.01	0.25 ± 0.01
Potassium (% DM)	0.27 ± 0.01	1.61	2.16	2.66 ± 0.18	0.81 ± 0.11	0.21 ± 0.08
TDN (% DM)	54.9 ± 1	50.1	52.5	57.7 ± 2	51.1 ± 1	54.8 ± 1
Predicted DMI (kg/d)^b^	14.0		13.7	13.4		13.1
ME available (Mcal/d)^b^	25.4		22.7	27.5		22.9
ME required (Mcal/d)^b^	23.9		24.3	22.9		24.4
MP available (g/d)^b^	410		369	442		372
MP required (g/d)^b^	541		531	556		559

a DM: Dry matter, NDF: Neutral detergent fiber, ADF: Acid detergent fiber, CP: Crude protein, TDN: Total digestible nutrients, DMI: Dry matter intake, ME: Metabolizable energy, MP: Metabolizable protein.

b Calculated using NASEM (2016).

### Animals and feeding

Thirty-six pregnant (artificially inseminated) Angus beef cows (*Bos taurus*) in the second trimester of gestation, averaging 709 ± 51 and 661 ± 62 kg (mean ± standard deviation) of initial body weight and 6.5 and 5.8 years of age were used for Trials 1 and 2, respectively. Each year, animals were divided into six homogenous groups based on body weight and housed in bedded pens measuring 7.3 × 24 m with 7.3 m of bunk at the Lancaster Agricultural Research Station. Diet treatments were offered *ad libitum* and allotted at random to each pen. A two-week adaptation period preceded the trials for both treatments. Diets were distributed daily with the mixing wagon at 1000 h, and the amount distributed was adjusted based on the previous day’s consumption. Forage allowance on the first day was 127 and 120 kg DM per pen in Trial 1 and 2, respectively, to be non-restrictive on forage intake, and it was reduced by 10 kg DM if residual in bunk exceeded 15 kg DM. Some material was wasted as the cows pulled some forage from the bunks onto the spoiled floor, which was estimated to represent 8% of the daily distributed forage for the 50-50 mix of haylage and Kernza straw diet. This behavior was not observed in the cows fed the 100% haylage diet.

Daily offered TMR, and orts from each pen were weighed daily. Pen intake was calculated as the difference between the offered ration and the refusals of the following day. Intake per cow was estimated by dividing the pen intake by the number of animals (6 per pen). Body weight measurements were taken at the beginning and end of the study using an electronic scale located outside the pens in the cattle handling facility. On the same days, body condition scores were assessed visually by two trained evaluators using a nine-point scale with 0.5-unit increments to best describe body condition according to NASEM (2016). Average daily gain was calculated as the difference between the final and initial bodyweight, divided by the trial duration (65 or 58 d).

### Statistical analyses

Statistical analyses were conducted with R version 4.1.2 (R Core Team, 2021). Pen was considered the experimental unit for all measurements. A one-way analysis of variance was performed using the following model:


$${\text{Y}}_{\text{ij}}=\;\mu \;+{\text{ Trt}}_{\text{i}}+{\text{ e}}_{\text{ij}},$$

where Y_ij_ are the response variables, μ is the overall mean, Trt_i_ is the fixed effect of the treatment, and e_ij_ is the random residual error. Significance was declared at alpha = 0.05. Estimated marginal means and standard error of the mean were calculated using the emmeans function (Lenth et al. 2024). Assumptions for the ANOVA, ie, the normal distribution of the residuals and the homogeneity of their variance, were tested by generating residual plots and confirming the randomness of the residuals.

## Results and discussion

Kernza straw did not affect DMI, whether as kg cow^−1 ^day^−1^ or as % of bodyweight, in Trial 1 (*P *> 0.05), but it was reduced in Trial 2 (*P *< 0.01, Table 2). It should be noted however that the DMI of cows fed the Kernza straw diet may have been overestimated because of greater feed bunk waste in those diets compared to the 100% haylage. The NASEM (2016) model predicted greater DMI than observed in both trials. Also, the difference between the predicted and the actual DMI was greater when cows were fed the Kernza straw diet than the 100% haylage diet in both trials. These results may indicate that the intake of cows fed the Kernza straw diet was in part limited by the level of NDF which was 6.4 units greater in the Kernza straw diet than the 100% haylage diet (66.9% vs 60.5% NDF) in Trial 1 and 12 units greater in Trial 2 (60.4% vs 48.4% NDF). Nevertheless, the average DMI observed for the cows fed 50% haylage and 50% Kernza straw was similar to the values reported for beef cattle consuming low-quality forages (Holder et al. 2022; Niederecker et al. 2018). Both groups of cows gained weight during the trials; however, the average daily gain for the cows eating 100% haylage (0.92 kg day^−1^) was greater than for those eating 50% Kernza straw and 50% haylage (0.41 kg day^−1^) in Trial 1 (*P *= 0.02). Cows in this study performed better than the NASEM (2016) model predicted; however, the model also predicted that all the diets were more limited by protein than by energy (Table 1). Body condition scores were not reduced in any trial, and the change in body condition scores was not affected by dietary treatment (*P *> 0.05). Cows in the present study were in optimal BCS (5 -7; Eversole et al. 2009); thus, their body fat reserves prevented to lose condition, despite of the reduction in intake or average daily gain. Additionally, we found no evidence of long-term negative effects on calf birth weights. In Trial 1, calves from cows fed 100% haylage weighed 39.5 kg at birth, while those from cows fed a 50-50 mix of haylage and Kernza straw weighed 40.6 kg. In Trial 2, birth weights were 41.8 kg for calves from cows fed 100% haylage, and 40.9 kg for those from cows fed the 50-50 mix. It is worth noting that these measurements were taken after the cows had been removed from the experimental diets. Specifically, calving occurred eleven weeks after the end of the study in Trial 1 and three weeks after in Trial 2.

**Table 2.** Performance of cows fed 100% grass-alfalfa haylage (*n* = 18) and 50% kernza straw and 50% grass-alfalfa haylage mixture (*n* = 18) at Lancaster, WI, USA over two years.

Parameters	Trial 1				Trial 2			
	Grass-alfalfa haylage diet	50-50 Kernza-haylage diet	SEM^a^	*P*-value^b^	Grass-alfalfa haylage diet	50-50 Kernza-haylage diet	SEM	*P*-value
Dry matter intake, kg cow^−1 ^day^−^	12.5	11.7	0.24	0.08	12.9	11.3	0.14	< 0.01
Dry matter intake, % of bodyweight	1.70	1.63	0.03	0.20	1.90	1.68	0.03	< 0.01
Initial bodyweight, kg	708	707	4.28	0.86	659	659	12.7	0.99
Final bodyweight, kg	767	734	9.73	0.07	701	684	11	0.33
Change in bodyweight, kg	58.8	26.5	6.22	0.02	42.1	25.0	6.26	0.13
Average daily gain, kg day^−1^	0.92	0.41	0.10	0.02	0.73	0.43	0.11	0.13
Initial body condition score	6.0	6.0	0.07	0.12	6.5	6.0	0.1	0.04
Final body condition score	6.5	6.0	0.07	0.03	7.0	6.5	0.1	0.04
Change in body condition score	0.5	0.0	0.08	0.31	0.5	0.5	0.05	0.70

a Standard error of the mean.

b Statistical significances were declared at *P *≤ 0.05.

The trial length might be considered a limitation of this study, as trials with a span of 90+ d remove some of the variability associated with bodyweight gain. However, studies have found that a minimum of 42 and 70 d are required to evaluate dry matter intake and average daily gain, respectively, in beef cattle (Ahlberg et al. 2018; Culbertson et al.2015; Marzocchi et al. 2020). In our case, the trial length was set due to the availability of resources, including Kernza straw, animals and facilities. Additionally, to avoid confounded effects by calving and lactation, we decided to have an eight to nine-week period during gestation. Nevertheless, this period may have been insufficient to observe the full effect of treatments on BCS. Thus, cautious interpretation of these data is warranted.

In this study, Kernza straw CP was higher than 4.8% and 2.6% DM reported by Hunter et al. (2020) and similar to 4.2% and 4.1% DM reported by Culman et al. (2023) for plants 1 and 3 years old, respectively. Similarly, straw fiber content found in this study was higher than 42.6% and 38.7% ADF and 70.2% and 68.6% NDF reported by Culman et al. (2023) for plants 1 and 3 years old, respectively. One reason for the high fiber content of the Kernza straw in this study was the threshing technique used to harvest grain. Indeed, the whole plants were swathed at a stubble height of 10 cm and threshed in a combine harvester once dried. This mechanical process causes a loss of leaves (Brink and Casler 2012). Direct harvesting the crop by raising the bar of the harvester to harvest the mature seed heads only and cutting the remaining stems and leaves similarly to a regular grass hay may reduce the loss of nutritive value during harvest (Favre et al. 2019; Pinto et al. 2022). This technique is particularly promising for intermediate wheatgrass stands intercropped with forage legumes to maximize forage nutritive value in the summer (Pinto et al. 2024).

Besides supplementation with a greater-quality forage, there are two other alternatives to utilize mature forages with insufficient CP for cows. The first alternative consists of supplementation with an N source, such as soybean meal or urea, which has been shown to increase forage intake and digestibility by increasing the metabolic activity and abundance of rumen microorganisms (Sanson et al. 1990; Stanton and Whittier 2006). The second alternative involves treatment with anhydrous ammonia and has equally positive impacts on dry matter intake and digestibility of mature forages and crop residues (Buettner 1978; Saenger et al. 1982).

Farmers in the US Midwest have reported that profitability is one of the main limitations for growing Kernza (Lanker et al. 2020). Moreover, the Kernza grain market is currently in a price discovery phase, with prices varying significantly based on the management system, year, region, and grain quality, and several authors (Hunter et al. 2020; Law et al. 2022; Pinto et al. 2022; Rodgers et al. 2025) agreed on the importance of straw on Kernza profitability. This importance was not due to its forage quality but to its yields, which Hunter et al. (2020) reported to be 3—4 times greater than hay (8.6 vs 2.5 Mg ha^−1^ on average for 3 years). Furthermore, Pinto et al. (2022) indicated that the subsidy provided by the Conservation Stewardship Program (NRCS-USDA 2024) played an important role in the profitability of Kernza intercropped with legumes in Wisconsin. Additionally, these authors found that the income from the Kernza grain covers the total costs of its production, leaving the Kernza straw as a zero-cost product. This result aligns with an online survey indicating that the primary motivation for farmers to adopt perennial grains is to maintain or enhance farm profitability (Wayman et al. 2019). Our results suggest that integrated crop-livestock systems present a promising opportunity for growing Kernza as a dual-use perennial grain and forage crop.

## Conclusions

Our results showed that the Kernza straw baled after grain harvest can be utilized by beef cattle when fed in equal proportion with a greater-quality forage. Cows fed Kernza straw maintained their condition. Given that mature, non-lactating beef cows do not need to gain weight during the winter unless their initial body condition score is lower than 5, a higher inclusion of Kernza straw may be able to maintain bodyweight; however, this hypothesis requires further research. Kernza straw used as forage for beef cows can contribute to the profitability of dual-use forage and perennial grain systems.

Conflicts of interest: The authors declare no conflict of interest.

## References

[txaf131-B1] Ahlberg C. M. et al. 2018. Test duration for water intake, ADG, and DMI in beef cattle. J. Anim. Sci. 96:3043–3054. 10.1093/jas/sky209. 29790937 PMC6095348

[txaf131-B2] AOAC International. 2000. Official methods of analysis. 17th ed. AOAC International, Arlington, VA.

[txaf131-B3] AOAC International. 2006. Official methods of analysis. 18th ed. AOAC International, Arlington, VA.

[txaf131-B4] Brink G. E. , Casler M. D. 2012. Yield and nutritive value differences among cool-season grasses. Forage and Grazinglands. 10:1–11. 10.1094/FG-2012-0619-01-RS.

[txaf131-B5] Buettner M. R. 1978. Effect of ammoniation on the composition and digestion of forage fiber [Ph.D. thesis]. Purdue University, West Lafayette, IN.

[txaf131-B6] Chapman E. A. et al. 2022. Perennials as future grain crops: opportunities and challenges. Front. Plant Sci. 13:898769. 10.3389/fpls.2022.898769. 35968139 PMC9372509

[txaf131-B7] Culbertson M. M. et al. 2015. Optimum measurement period for evaluating feed intake traits in beef cattle. J. Anim. Sci. 93:2482–2487. 10.2527/jas.2014-8364. 26020343

[txaf131-B8] Culman S et al. 2023. Forage harvest management impacts “kernza” intermediate wheatgrass productivity across North America. Agron. J. 115:2424–2438. 10.1002/agj2.21402.

[txaf131-B9] Eversole , Browne D. M., Hall J., Dietz R. 2009. Body condition scoring beef cows. Virginia Cooperative Extension Publication, 400–791. [Accessed May 2, 2025]. https://www.vabeginningfarmer.alce.vt.edu/content/dam/vabeginningfarmer_alce_vt_edu/taste-of-farming/beefcattle/BodyConditionScoringBeefCows.pdf

[txaf131-B10] Favre J. R. , Munoz T., Combs D. K., Wattiaux M. A., Picasso V. D. 2019. Forage nutritive value and predicted fiber digestibility of kernza intermediate wheatgrass in monoculture and in mixture with red clover during the first production year. Anim. Feed. Sci. Technol. 258:114298. 10.1016/j.anifeedsci.2019.114298.

[txaf131-B11] Franco J. G. et al. 2021. Ecological intensification of food production by integrating forages. Agronomy. 11:2580. 10.3390/agronomy11122580.

[txaf131-B12] Holder A. L. et al. 2022. Effects of diet on feed intake, weight change, and gas emissions in beef cows. J. Anim. Sci. 100:skac257. 10.1093/jas/skac257. 35952719 PMC9527298

[txaf131-B13] Hunter M. C. , Sheaffer C. C., Culman S. W., Lazarus W. F., Jungers J. M. 2020. Effects of defoliation and row spacing on intermediate wheatgrass II: Forage yield and economics. Agron. J. 112:1862–1880. 10.1002/agj2.20124.

[txaf131-B14] Lanker M. , Bell M., Picasso V. 2020. Farmer perspectives and experiences introducing the novel perennial grain kernza intermediate wheatgrass in the US midwest. Renew. Agric. Food Syst. 35:653–662. 10.1017/S1742170519000310.

[txaf131-B15] Law E. P. et al. 2022. Multi-Criteria assessment of the economic and environmental sustainability characteristics of intermediate wheatgrass grown as a Dual-Purpose grain and forage crop. Sustainability. 14:3548. 10.3390/su14063548.

[txaf131-B16] Lenth R. et al. 2024. Emmeans: Estimated marginal means, aka Least-Squares means. R Package Version 1.10.2 Computer Software. 10.32614/CRAN.package.emmeans

[txaf131-B17] Linn J. G. , Martin N. P. 1989. Forage quality tests and interpretation. Minn. Ext. Serv. AG-FO-2637. Univ. of Minn., Saint Paul. [Accessed May 2, 2025]. https://conservancy.umn.edu/server/api/core/bitstreams/8ac6e7dc-4bb0-4d5f-881f-8ff20d04a5e3/content

[txaf131-B18] Marzocchi M. Z. , Sakamoto L. S., Canesin R. C., Gonçalves Cyrillo J. D. S., Zerlotti Mercadante M. E. 2020. Evaluation of test duration for feed efficiency in growing beef cattle. Trop. Anim. Health Prod. 52:1533–1539. 10.1007/s11250-019-02161-0. 31813088

[txaf131-B19] Moore J. E. , Undersander D. J. 2001. Relative forage quality: an alternative to relative feed value and quality index. Proceedings of the 13th Annual Florida Ruminant Nutrition Symposium, pp. 16–32, January 10-11, 2002, Gainsville, FL, USA.

[txaf131-B20] NASEM. 2016. Nutrient requirements of beef cattle: Eighth revised edition. The National Academies Press, Washington, DC. 10.17226/19014 38386771

[txaf131-B21] NFTA. 2001. Moisture task force report. National Forage Testing Association, Omaha, NE

[txaf131-B22] NRCS-USDA. 2024. Conservation Stewardship Program-Wisconsin-E328O. [Accessed September 18, 2024]. https://www.nrcs.usda.gov/wps/PA_NRCSConsumption/

[txaf131-B23] Niederecker K. N. , Larson J. M., Kallenbach R. L., Meyer A. M. 2018. Effects of feeding stockpiled tall fescue versus summer-baled tall fescue-based hay to late gestation beef cows: I. Cow performance, maternal metabolic status, and fetal growth. J. Anim. Sci. 96:4618–4632. 10.1093/jas/sky341. 30137366 PMC6247859

[txaf131-B24] Picasso V. D. et al. 2022. Diverse perennial circular forage systems are needed to foster resilience, ecosystem services, and socioeconomic benefits in agricultural landscapes. Grassland Res. 1:123–130. 10.1002/glr2.12020.

[txaf131-B25] Pinto P. et al. 2022. Intercropping legumes and intermediate wheatgrass increases forage yield, nutritive value, and profitability without reducing grain yields. Front. Sustain. Food Syst. 6:977841. 10.3389/fsufs.2022.977841.

[txaf131-B26] Pinto P. , Cartoni-Casamitjana S., Stoltenberg D. E., Picasso V. D. 2024. Forage boost or grain blues? Legume choices shape kernza intermediate wheatgrass dual-purpose crop performance. Field Crops Res. 316:109522. 10.1016/j.fcr.2024.109522.

[txaf131-B27] Rodgers , Fancher H. H., Foulke T., Peters T. 2025. Kernza^®^ perennial grain and wheat-fallow budgets: comparing a perennial and annual cropping system in Southeastern Wyoming. University of Wyoming Extension. [Accessed August 20, 2025]. https://www.wyoextension.org/agpubs/pubs/B-1403.pdf

[txaf131-B28] Saenger P. F. , Lemenager R. P., Hendrix K. S. 1982. Anhydrous ammonia treatment of corn stover and its effects on digestibility, intake and performance of beef cattle. J. Anim. Sci. 54:419–425. 10.2527/jas1982.542419x.

[txaf131-B29] Sanson D. W. , Clanton D. C., Rush I. G. 1990. Intake and digestion of low quality meadow hay by steers and performance of cows on native range when fed protein supplements containing various levels of corn. J. Anim. Sci. 68:595–603. 10.2527/1990.683595x. 2318726

[txaf131-B30] Stanton T. L. , Whittier J. 2006. Urea and NPN for cattle and sheep. Fact sheet no. 1608. Colorado State University Extension. [Accessed May 2, 2025]. https://extension.colostate.edu/docs/pubs/livestk/01608.pdf

[txaf131-B31] Stewart W. M. , Dibb D. W., Johnston A. E., Smyth T. J. 2005. The contribution of commercial fertilizer nutrients to food production. Agron. J. 97:1–6. 10.2134/agronj2005.0001.

[txaf131-B32] The Land Institute. 2024. The State of Kernza^®^. [Accessed May 2, 2025]. https://kernza.org/the-state-of-kernza/

[txaf131-B33] Tilman D. , Cassman K. G., Matson P. A., Naylor R., Polasky S. 2002. Agricultural sustainability and intensive production practices. Nature. 418:671–677. 10.1038/nature01014. 12167873

[txaf131-B34] Titgemeyer E. C. et al. 2004. Effect of forage quality on digestion and performance responses of cattle to supplementation with cooked molasses blocks. J. Anim. Sci. 82:487–494. 10.2527/2004.822487x. 14974547

[txaf131-B35] Wayman S. , Debray V., Parry S., David C., Ryan M. R. 2019. Perspectives on perennial grain crop production among organic and conventional farmers in France and the United States. Agriculture. 9:244. 10.3390/agriculture9110244.

[txaf131-B36] USDA. 2022. Beef cattle producers face higher input costs, with feed prices up 16 percent since 2021. Economic Research Service. [Accessed May 2, 2025]. https://www.ers.usda.gov/data-products/charts-of-note/chart-detail? chartId=104424

